# Prevalence of Prehypertension Among Adults in Baghdad/Iraq

**DOI:** 10.1155/ijhy/2659005

**Published:** 2026-04-28

**Authors:** Methaq H. Alogaili, Afnan A. Alsallami, Mustafa S. Fadhil, Sadeq H. Khaleel

**Affiliations:** ^1^ Department of Family and Community Medicine, College of Medicine, Al-Nahrain University, Baghdad, Iraq, nahrainuniv.edu.iq; ^2^ College of Medicine, Al-Nahrain University, Baghdad, Iraq, nahrainuniv.edu.iq; ^3^ University of Lancashire School of Medicine and Dentistry, Preston, UK

**Keywords:** blood pressure, cross-sectional study, prehypertension, prevalence.public health

## Abstract

**Introduction/Objective:**

Prehypertension (defined as systolic pressure of 120–139 mmHg or diastolic pressure of 80–89 mmHg) may impose a substantial burden and future public health challenge. Prehypertension is associated with a high risk of progression to hypertension and subsequent cardiovascular complications, with a 40% five‐years progression rate. In those people, lowering blood pressure helps prevent progression to frank hypertension and subsequent target organ damage. In Iraq, the prevalence of hypertension is about 35.6%, and the blood pressure in one‐third was uncontrolled. To our knowledge, there are no studies addressing prehypertension in Iraq. This study aimed to assess the prevalence of prehypertension among adults in Baghdad/Iraq.

**Methods:**

This cross‐sectional study enrolled 424 adults (18 years old and above) recruited from randomly selected primary healthcare centers distributed throughout the city of Baghdad (capital of Iraq) using a multistage sampling technique. Blood pressure was measured (two readings were taken 10‐min apart, and the mean value for both systolic and diastolic readings was calculated in millimeters of mercury). Those with a history of hypertension, on antihypertensive treatment or pregnant women were excluded from the study.

**Results:**

The prevalence of prehypertension was 31.0%. There was a significant difference in the preHTN group between normal and high waist circumference (32.1% vs. 49.0%, *p* = 0.04). Predicted mean blood pressure was significantly correlated with both body mass index and waist circumference (*p* < 0.001, R‐squared = 0.03). The adjusted multiple logistic regression model shows a significant association of prehypertension with male sex (OR = 2.72, CI = 1.65 to 6.41), high fasting glucose, and high cholesterol level.

**Conclusions:**

Prehypertension (as well as risk factors) is highly prevalent among Iraqi adults. Targeted screening programs, lifestyle modifications, and more focus are needed.

## 1. Background

Prehypertension (PreHTN), an intermediate stage between normal blood pressure and hypertension (HTN), is defined as a systolic blood pressure of 130–139 mm of mercury (mmHg) and/or a diastolic blood pressure of 80–89 mmHg [1]. PreHTN and HTN present significant clinical and public health challenges in both developing and developed nations. They are associated with subclinical atherosclerosis, target organ damage, and a 10% absolute risk of cardiovascular disease (CVD) over 10 years for middle‐aged adults without diabetes mellitus or CVD, rising to 40% for middle‐aged and older individuals with either or both comorbidities [2].

PreHTN affects approximately 25%–50% of adults globally. However, due to its silent nature, the prevalence is underreported, particularly in low‐ and middle‐income countries [3]. It increases the risks of progression to HTN, cognitive function impairment, increased left ventricular mass, risk of end‐stage renal disease, and arteriosclerosis [4]. Among young adults worldwide, the incidence of preHTN ranges from 37.5% to 77.1% [5].

Time is a critical factor in addressing silent conditions such as preHTN, which are linked to progressive arteriosclerosis and a host of cardiovascular complications arising from uncontrolled elevated blood pressure [6]. Compared to normal blood pressure, PreHTN carries a higher risk of progression to HTN and cardiovascular events, with a 5‐year progression rate of 40%. Lifestyle modifications in individuals with PreHTN can significantly reduce blood pressure, preventing progression to HTN and mitigating target organ damage and CVD risk [7].

In Iraq, the prevalence of HTN is reported to be 35.6%, with one‐third of cases uncontrolled. In addition, the prevalence of risk factors for noncommunicable diseases is alarmingly high [8]. However, to the best of our knowledge, no studies have been conducted addressing the prevalence of PreHTN in Iraq.

## 2. Objectives

This study aimed to estimate the prevalence of PreHTN among adults in Baghdad, Iraq, and to assess some sociodemographic characteristics and risk factors in this population.

## 3. Methods

### 3.1. Study Setting, Study Design, and Study Participants

This is a cross‐sectional study conducted in the capital city of Iraq, Baghdad, for a period of three months starting from February 2024. Using a multistage sampling technique, the study participants were recruited from randomly selected primary healthcare centers that were dispersed equally over the whole city. The sample size was calculated using the single‐proportion formula, and 10% were added as a nonresponse rate, yielding a total sample of 424 participants. The study included all adults (aged more than 18 years) with no self‐reported history of HTN on antihypertensive treatment or pregnant women. Participants were directly interviewed, and the needed demographic characteristics were filled in a specifically constructed questionnaire. Demographic characteristics included age, sex, marital status, education, occupation, smoking history, alcohol intake, and past medical history.

### 3.2. Study Variables

Weight and height were measured to calculate body mass index (BMI) (kg/m^2^) and waist circumference (WC), which were used as determinants of abdominal obesity and were measured according to WHO criteria [9]. Blood pressure was measured (two readings were taken 10‐min apart, and the mean value for both systolic and diastolic readings was calculated in millimeters of mercury) according to the international recommendations [10]. PreHTN was defined as systolic pressure of 120–139 mmHg or diastolic pressure of 80–89 mmHg [11]. Blood was drawn (2 mL) to measure blood sugar, total cholesterol, triglycerides, and complete blood count.

### 3.3. Statistical Analysis

Data were analyzed using SPSS Version 26. Mean ± standard deviation was used to present continuous variables, and an independent *t*‐test was used to compare the mean differences between groups. Categorical variables were expressed as counts and frequencies, and the chi‐square test was used to test the association between groups. A binary logistic regression model was used to test the association of independent variables with the outcome variable (preHTN or not); WC and TG were excluded from the model as there was no linear relationship found with the outcome. The Pearson correlation coefficient was used to evaluate the association between BMI and WC in both males and females. Multiple linear correlation was used to predict mean blood pressure by WC and BMI. A *p* value less than 0.05 was considered statistically significant.

## 4. Results

**FIGURE 1:**
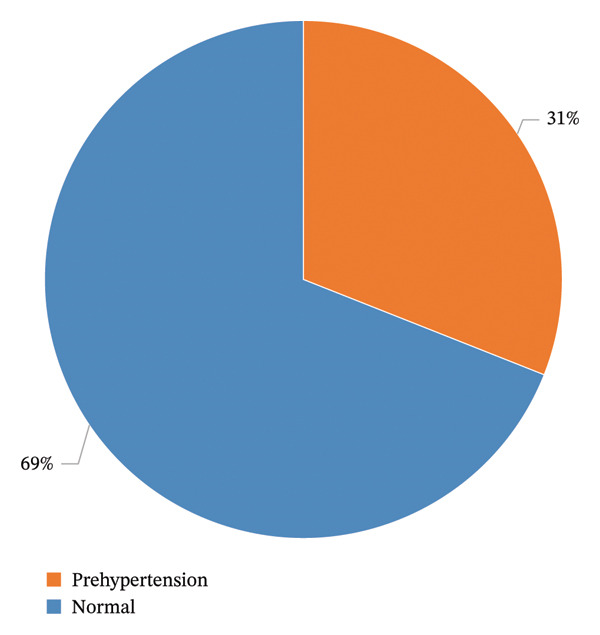
Prevalence of preHTN.

Fifty‐three participants were found to be hypertensive and were excluded from the study. The prevalence of preHTN was 31.0% (Figure 1).

Table 1: Of the study participants (*n* = 371), the mean age was 42.9 ± 13.5 (range 20–70 years), and 57.7% were females. The mean BMI and WC were 27.6 ± 4.7 and 87.8 ± 13.8, respectively.

**TABLE 1:** Characteristics of the study population.

Variables	Overall	Prehypertension	Normal	*p* value
*N*	371	115	256	
Age (years)	42.9 ± 13.5	45.5 ± 13.6	41.8 ± 13.4	***p* = 0.02**
20–44	205 (55.3%)	51 (24.9%)	154 (75.1%)	***p* = 0.01**
45–70	166 (44.7%)	64 (38.6%)	102 (61.4%)	
Sex				
Female	214 (57.7%)	47 (22.0%)	167 (78.0%)	***p* < 0.001**
Male	157 (42.3%)	68 (43.3%)	89 (56.7%)	***p* < 0.001**
BMI (kg/m^2^)	27.5 ± 4.7	28.8 ± 4.7	26.9 ± 4.6	***p* < 0.001**
Normal	116 (31.3%)	29 (25.0%)	87 (75.0%)	***p* = 0.04**
Overweight	154 (41.5%)	45 (29.2%)	109 (70.8%)	
Obese	101 (27.2%)	41 (40.6%)	60 (59.4%)	
Waist circumference (cm)				
Female	83.6 ± 11.4	89.3 ± 10.0	82.0 ± 11.3	***p* < 0.001**
Male	97.2 ± 13.3	99.8 ± 12.3	95.3 ± 13.8	***p* = 0.03**
Average SBP (mmHg)	121.6 ± 11.3	130.0 ± 9.1	117.9 ± 10.1	***p* < 0.001**
Average DBP (mmHg)	78.1 ± 8.5	82.5 ± 6.8	76.2 ± 8.5	***p* < 0.001**
FBG (mg/dL)	111.7 ± 39.1	118.5 ± 37.5	108.7 ± 39.5	***p* = 0.02**
Cholesterol (mg/dL)	193.2 ± 39.7	203.8 ± 43.4	188.4 ± 37.0	***p* = 0.001**
TG (mg/dL)	159.6 ± 62.3	186.7 ± 70.0	147.5 ± 54.3	***p* < 0.001**
Diabetes	101 (27.2%)	41 (40.6%)	60 (59.4%)	***p* = 0.02**
Alcohol	30 (8.1%)	10 (33.3%)	20 (66.7%)	*p* = 0.80
Smoking				***p* = 0.03**
Yes	114 (30.7%)	41 (36.0%	73 (64.0%)	
No	230 (62.0%)	61 (26.5%)	169 (73.5%)	
Ex‐smoker	27 (7.3%)	13 (48.1%)	14 (51.9%)	
Marital status				*p* = 0.36
Unmarried	53 (14.3%)	12 (22.6%)	41 (77.4%)	
Currently married	254 (68.5%)	82 (32.3%)	172 (67.7%)	
Divorced or widow	64 (17.3%)	21 (32.8%)	43 (67.2%)	
Education				*p* = 0.73
Illiterate	51 (13.7%)	18 (35.3%)	33 (64.7%)	
Secondary and less	232 (62.5%)	69 (29.7%)	163 (70.3%)	
Higher education	88 (23.7%)	28 (31.8%)	60 (68.2%)	
Occupation				***p* = 0.02**
Employed	198 (53.3%)	74 (37.4%)	124 (76.5%)	
Non	136 (36.7%)	32 (23.5%)	104 (40.6.8%)	
Retired	37 (10.0%)	9 (24.3%)	28 (75.7%)	

*Note:* Bold values indicate statistically significant differences (*p* < 0.05) between the prehypertension and normal groups.

In the preHTN group (*n* = 115), the mean age was 45.5 ± 13.6 (range 21–70 years), and 59.1% were males. The mean BMI was 28.8 ± 4.7, and the mean WC was 93.3 ± 13.4 cm. The mean age of the preHTN group was significantly higher (*p* = 0.02), while no significant association was found in different age classes between the two groups (*p* = 0.13). There was a significant difference in sex between the two groups (*p* < 0.001).

People with preHTN were significantly different in the mean of most parameters (BMI, WC, FBG, cholesterol, TG, smoking, and diabetes) than those with normal BP.

No significant association was found with marital status and education level.

Table 2: Regarding WC cutoff points for risk of metabolic complications, there was a significant difference in the preHTN group between normal and high WC (32.1% vs 49.0%, *p* = 0.04). This significant association was also found in females (32.1% vs. 49.0%, *p* = 0.002).

**TABLE 2:** Waist circumference cutoff points of the study population for metabolic complications.

Variable		Prehypertension	Normal	*p* value
Male	Normal	17 (32.1%)	36 (67.9%)	***p* = 0.04**
	≥ 94 cm	51 (49.0%)	53 (51.0%)	

Female	Normal	8 (10.3%)	70 (89.7%)	***p* = 0.002^∗^**
	≥ 80 cm	39 (28.7%)	97 (71.3%)	

*Note:* Bold values indicate statistically significant differences (*p* < 0.05) between the prehypertension and normal groups.

^∗^Fisher’s exact test.

Table 3: Multiple logistic regression analyses were performed to assess the association between the study groups′ outcome (preHTN or not), sociodemographic characteristics, and anthropometric measurements. First, separate models for each independent variable of interest were analyzed. Thereafter, variables included in an adjusted model, except for WC and TG, were excluded as there was no linear relationship found with the outcome. Alcohol was also eliminated from the adjusted model as the validity of self‐reported alcohol intake was considered uncertain.

**TABLE 3:** Multiple logistic regression to assess the association between study groups and participants’ characteristics (*n* = 371).

Variables	Univariate			Adjusted model		
	OR	95% CI	*p* value	OR	95% CI	*p* value
Age	1.02	1.00 to 1.03	0.02	1.02	0.99 to 1.04	0.20
Sex						
Female	ref					
Male	2.72	1.72 to 4.23	< 0.001	3.25	1.65 to 6.41	**0.001**
BMI class						
Normal	ref					
Overweight	1.24	0.72 to 2.14	0.44	1.08	0.59 to 1.98	0.80
Obese	2.05	1.15 to 3.66	0.02	1.80	0.92 to 3.51	0.08
FBG (mg/dL)	1.01	1.00 to 1.01	0.03	1.01	1.00 to 1.01	**0.04**
Cholesterol (mg/dL)	1.01	1.00 to 1.02	0.001	1.01	1.00 to 1.01	**0.02**
Diabetes						
No	ref					
Yes	1.81	1.12 to 2.92	0.02	1.44	0.81 to 2.58	0.22
Smoking						
No	ref					
Yes	1.56	0.96 to 2.52	0.72	0.89	0.48 to 1.65	0.72
Ex‐smoker	2.57	1.12 to 5.78	0.22	1.61	0.58 to 4.49	0.36
Marital status						
Unmarried	ref					
Currently married	1.63	0.81 to 3.26	0.17	1.01	0.46 to 2.25	0.98
Divorced or widow	1.67	0.73 to 3.82	0.23	1.85	0.72 to 4.77	0.20
Education						
Illiterate	ref					
Secondary or less	0.78	0.41 to 1.47	0.45	0.87	0.45 to 1.97	0.87
Higher education	0.86	0.41 to 1.77	0.68	0.94	0.41 to 2.14	0.87
Occupation						
Retired	ref					
Nonemployed	0.96	0.41 to 2.24	0.92	3.49	1.17 to 10.38	**0.03**
Employed	1.32	0.83 to 4.15	0.13	4.28	1.54 to 11.95	**0.005**

*Note:* Bold values indicate statistically significant results at the threshold of *p* < 0.05.

The adjusted regression model shows a significant association of preHTN with sex (being male), high FBG, cholesterol, nonemployment and, being employed.

**FIGURE 2:**
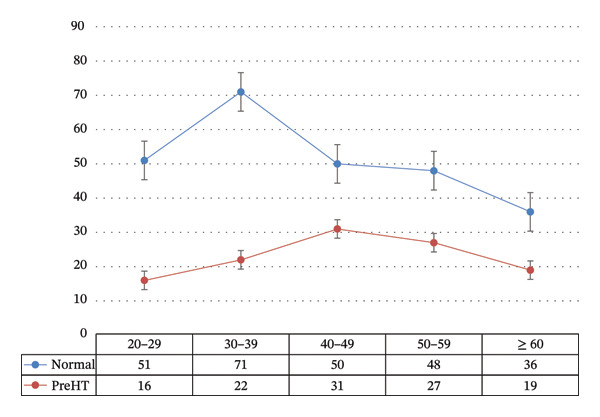
Blood pressure categories by age groups.

Figure 2: Higher rates of preHTN in the active age group (30–60) and then a decline, which may represent conversion to frank HTN.

**FIGURE 3:**
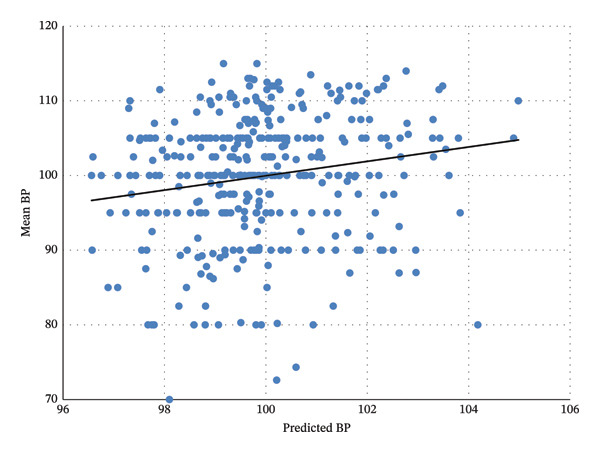
Predicted mean blood pressure by predicted values of both waist circumference and BMI (*p* < 0.001).

Figure 3: Predicted mean BP was significantly correlated with predicted values of both BMI and WC (*p* < 0.001, *R* ^2^ = 0.03).

**FIGURE 4:**
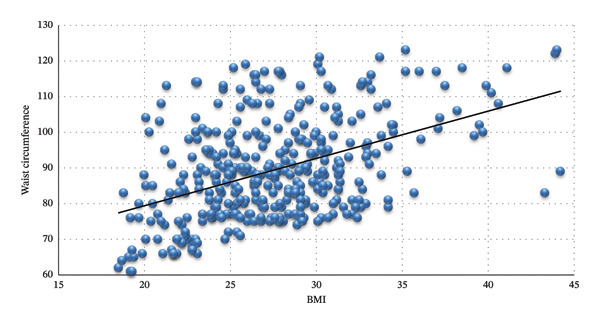
Correlation between BMI and waist circumference (*r* = 0.45, *p* < 0.001).

Figure 4 shows a significant good correlation between BMI and WC (*r* = 0.45, *p* < 0.001).

## 5. Discussion

Addressing all aspects of human health, particularly conditions that are asymptomatic or have hidden pathophysiology, is essential in healthcare research. Such conditions can lead to adverse effects if left unrecognized. Studying the prevalence of these conditions, in addition to being a fundamental epidemiological concept, provides valuable data with a significant impact on healthcare systems. The prevalence of preHTN and HTN is expected to rise in the upcoming years, with the connections to numerous other cardiovascular and noncardiovascular health conditions, different treatment options, management protocols, and prevention strategies, which places a significant pressure on the health system [12]. This growing burden emphasizes the importance of continued research and monitoring trends. PreHTN should be considered as a valuable tool in predicting cardiovascular health and implementing preventive strategies, as it is possible to restore normal blood pressure with diet and lifestyle modifications.

In this study, 31% of the population was identified as having preHTN, aligning with global prevalence reported in other studies ranging from 22% to 38% [13, 14]. In neighboring countries, a 2022 study showed that the prevalence of preHT among Turkish adolescents was 11.2% [15], while in Kuwait, it was 39.5% among college students [16].

Rahut DB et al. studied the prevalence of preHTN among adults (18–49 years) of South Asia and concluded that about one‐third of the study population had preHTN. In addition, a higher preHTN to HTN ratio is observed [17].

In this study, WC was significantly higher in the preHTN group and in both sexes. Furthermore, a significant association between WC cutoff points for metabolic complications was also noted for both sexes with the preHTN group. This finding is also supported by a cohort study conducted on US adults [18]. In another cohort study performed by Qin P et al., WC increment was regarded as a predictor for the progression from preHTN to HTN [19] (Figure 3).

Generally, there are two important metrics with a determined optimal range of body weight normality for sex and age: BMI and WC. Both measures are related to human cardiovascular health and metabolic status. BMI combines weight and height to assess body fat and potential health risks, while WC is specifically related to abdominal obesity. WC is one of the clinical parameters related to blood pressure, BMI, on the other hand, is also related but to a less specific degree when compared to WC as its value does not directly reflect the central obesity nor it specifies the fat mass as both play a role in cardiometabolic risk prediction and signify a poor prognosis [20] (Figure 4).

Waist‐to‐hip ratio is another measure of central adiposity, which also correlates with cardiovascular risk. However, WC is still the measure most closely associated with abdominal fat mass and, consequently, cardiovascular risk [21].

Analysis and comparison between both sexes showed that the relationship between WC and preHTN is more evident in males; this fact may be attributed to several gender related biological and metabolic factors. A study showed that a higher WC in normal‐BMI men with coronary artery disease is associated with more severe coronary artery injury and higher one‐year readmission rates, while this relationship is absent in women [22].

The multiple logistic model showed that male sex is a predictor of preHTN. This is in agreement with other studies [23, 24]. However, some researchers suggest varying gender disparities due to differences in androgen levels between biological males and females [25].

This study′s results confirmed the direct relationship between obesity (indicated by high BMI) and high blood pressure as well as other indicators of metabolic syndrome such as high cholesterol and high FBG. Adipose tissue, especially in the abdominal area, secretes inflammatory cytokines that can lead to endothelial dysfunction and increased arterial stiffness, thus raising blood pressure.

Visceral adiposity also contributes in another way to the process, as studies have shown that the accumulation of fat around the kidneys raises the intra‐abdominal and intra‐renal pressures, disturbing renal regulation, which is related to the direct physiological relationship between renin–angiotensin–aldosterone system (RAAS) and high blood pressure [26].

Higher hemoglobin (Hb) and hematocrit levels showed a positive association with preHTN, falling within the normal reference range for both sexes. Studies have shown that increased levels of these indicators are associated with vasoconstriction and the subsequent elevation of the systolic and diastolic blood pressures [27]. This positive correlation has been reported by other studies for both HTN itself and preHTN in otherwise healthy individuals [28, 29].

The majority of preHTN cases affect individuals aged between 30 and 50 years old. However, preHTN was seen among all these study age groups (Figure 2). Looking at the statistics from another study conducted on the adolescent group, 16.8% of them were shown to have preHTN [30]. Seemingly, the age range affected by preHTN is expanding, and although this expansion may be attributed to recent increased focus and surveillance, it revealed much about the current health status and actual steps that should be taken by healthcare facilities. preHTN shares the same risk factors in adolescents as in adults, among them, high BMI is the most important risk factor for this age group [31, 32].

In individuals diagnosed with Type 2 diabetes (T2DM), lower systolic and diastolic blood pressures are favorable, along with early initiation of treatment for any elevation in blood pressure above 130/80 mm·Hg. Therefore, diabetic preHTN is defined as systolic BP of 110–129 mmHg and/or diastolic BP of 70–79 mmHg [33]. Nondiabetic preHTN is considered HTN in T2DM, requiring treatment with antihypertensive medications. These guidelines were developed following the established association between intensive blood pressure control and a reduced risk of stroke and other macro‐ and microvascular complications associated with T2DM [34]. Another study suggested initial management with 3‐months of lifestyle changes prior to the initiation of pharmacological therapy in diabetic PreHTN individuals [35]. It is worth noting that the above definitions also apply to PreHTN individuals with metabolic syndrome [36].

In this study, a statistically significant inverse relationship between preHTN and smoking was observed. Smoking is a traditional risk factor for HTN in the literature. However, the relationship between smoking and blood pressure seems complicated; many studies showed different opinions and physiological explanations. Factors such as the BP‐lowering effect of nicotine or the effect of smoking on lipid profile, with the possibility of subsequent atherosclerosis, explain the low or high blood pressure in smokers, respectively. Furthermore, there is a group of smokers whose blood pressure shows no alteration with smoking [37, 38]. It is worth noting that these results related to smoking (and alcohol) are in alignment with other studies conducted in Iraq and were always explained by cultural and religious self‐underreporting of these factors [39].

Of the study limitations, as for any cross‐sectional study, conclusions and inferences cannot be made; some sociodemographic characteristics were self‐reported, and the study was limited to one city of the country. Nevertheless, the study settings and population coverage can allow generalization of the results.

## 6. Conclusion

PreHTN is prevalent among Iraqi adults with a high frequency of metabolic risk factors. Addressing lifestyle modifications, screening programs, community engagement, and education and encouraging public health initiatives can mitigate the progression to HTN and reduce future complications.

## Funding

No financial support has been provided for this study.

## Ethics Statement

The study was approved by the institutional review board (IRB) of the College of Medicine, Al‐Nahrain University. All participants were informed about the research purpose, and the confidentiality of patients’ information was ensured.

## Conflicts of Interest

The authors declare no conflicts of interest.

## Data Availability

The data that support the findings of this study are available on request from the corresponding author. The data are not publicly available due to privacy or ethical restrictions.
